# Advocating for child health through storytelling

**DOI:** 10.1038/s41390-025-04198-4

**Published:** 2025-06-05

**Authors:** Dawn Gano

**Affiliations:** Departments of Neurology and Pediatrics, University of California, San Francisco, San Francisco, CA USA

The return on investment in pediatric research is the profound impact for individual children, and families, as well as society at large.^1^ Although pediatric research funding has been ostensibly stagnant,^2^ we have accomplished tremendous gains as reflected by improving life expectancy. Notable achievements in pediatric research were highlighted by Cheng et al.^3^ in this Journal nearly a decade ago, including the prevention of infections through life-saving immunizations, reducing sudden infant death with back to sleep, making acute lymphoblastic leukemia curable, reducing mortality in preterm infants from respiratory distress syndrome, increasing life expectancy of chronic childhood diseases like sickle cell anemia and cystic fibrosis, preventing perinatal human immunodeficiency virus transmission, as well as implementing safety measures like car seats and seatbelts. From policy to precision medicine, pediatric research has a demonstrable track record of persistence and progress.

Using the illustrative examples of hyperbilirubinemia and airline safety for children under 2 years, the inimitable pediatrician and epidemiologist, Dr. Thomas Newman, persuasively argued for the power of stories over statistics in a commentary in 2003.^4^ While effective public policy is based upon evidence, Dr. Newman argued that policy is crafted and enacted by people, and people are more likely to be compelled by narratives than numbers.^4^ I believe pediatric researchers urgently need to tell more stories *now* to promote child health.

As a pediatric neurologist, my work is devoted to promoting neurological health in utero and across the life cycle. Walking parents and children through difficult diagnoses, developing treatment plans with families, and navigating uncertainty in the face of serious neurological illness demands compassion and conviction. This work requires an inherent sense of hope that no matter how the path ahead may unfold, we can improve the lives of our patients and their families through partnership in clinical care. My hope also stems from the promise of pediatric research.

The incredible potential of novel therapies in the pediatric population is now being realized across disciplines. Particular progress has been made in the field of neurology, a specialty once notorious for the lack of therapies beyond supportive care. We have seen therapeutic hypothermia for neonatal encephalopathy due to probable perinatal hypoxia-ischemia transform the landscape to improve outcomes.^5,6^ We have seen revolutionary therapies for spinal muscular atrophy (SMA) completely change the trajectory of disease.^7,8^ Whereas many forms of SMA were progressively degenerative and fatal in my training as a pediatric neurologist, the tenacity of an untold number of scientists and clinicians has culminated in numerous breakthroughs to convert this to a treatable condition. Many other examples of the hope of novel therapeutic approaches abound in neurology and throughout pediatrics.^9^

We must acknowledge that the hope for research to improve patient outcomes in the future is now under threat by the dismantling of scientific funding and infrastructure that is presently underway. So too are the early intervention services and learning supports that many of our patients and their families rely on: Head Start, Individualized Education Programs administered through local school districts, as well as policies to ameliorate child poverty. Moreover, proposed cuts to federal Medicaid spending would limit access to healthcare for millions of children and adults. Like elephants that stand shoulder to shoulder to protect their calves during an earthquake, we must encircle our vulnerable patients and families to protect the best possible outcomes for their futures through the preservation of pediatric research, and the progress we have made.

Storytelling can strategically communicate the stakes of pediatric research and cut through the noise. This includes telling stories about what we do and why, as well as our patients’ stories. I write this commentary to fulfill a promise I made to my patient’s parents. “Cece” was born at 29 weeks’ gestation and I first met her when she was diagnosed with intraventricular hemorrhage and periventricular hemorrhagic infarction shortly after birth. Now 1.5 years old, she is among the happiest infants I have met, and also among the most determined. Her parents recently shared their worry about how the potential closure of the United States Department of Education could impact Cece’s future access to school-based supports, therapies, and accommodations. I reiterated a commitment I first made to them when she was one week old: to use my voice as an advocate for children of all abilities, and to promote improved outcomes for children with perinatal brain injury and cerebral palsy through research.

I have made a version of this same commitment to countless families in countless ways, recognizing the nuance in every child and family’s story. For instance, one of my patients last week was a 5-year old child with prior perinatal hypoxic-ischemic encephalopathy and cerebral palsy, and an aptitude for elaborate freehand Lego builds. The parents reported that since our last visit, he had a neuropsychological evaluation that identified features of autism spectrum disorder. They expressed worry about this information being in his medical chart in light of reported plans for a national registry of individuals with autism spectrum disorder. While this child is privately insured and would not be captured in the proposed national database, my patients with public insurance would be. One such Kindergarten-aged girl with autism and cerebral palsy also came to see me last week. I have been following her since she was born at 24 weeks’ gestation. She had developed new episodes that she called “the shakies,” the onset of which coincided with her being bullied at school, with some of her peers taunting her about being sent to Mexico. Meeting this moment requires us to tell these stories.

Children deserve fair opportunities to learn and thrive - public policy and research are central to this goal. In the current information ecosystem, we need the narrative of pediatric researchers to reflect our reality: who we are, what drives our mission to improve child health, what our studies show and mean, and how defunding our work will impact children, families, and communities. Mastering the skillset of storytelling and engaging the broader public are essential to further child health in the modern era. I encourage pediatric researchers at all career levels to seize every possible opportunity for public engagement to protect child health and pediatric research. Let us stand shoulder to shoulder together.
